# Preprocedural and procedural variables that predict new-onset conduction disturbances after transcatheter aortic valve replacement

**DOI:** 10.1186/s12872-022-02576-y

**Published:** 2022-03-31

**Authors:** Wongsaput Boonyakiatwattana, Adisak Maneesai, Vithaya Chaithiraphan, Decho Jakrapanichakul, Pranya Sakiyalak, Narathip Chunhamaneewat, Worawong Slisatkorn, Chunhakasem Chotinaiwattarakul, Rungtiwa Pongakasira, Nattawut Wongpraparut

**Affiliations:** 1 Division of Cardiology, Department of Medicine, Faculty of Medicine Siriraj Hospital, Mahidol University, 2 Wanglang Road, Bangkoknoi, Bangkok, 10700 Thailand; 2 Division of Cardiothoracic Surgery, Department of Surgery, Faculty of Medicine Siriraj Hospital, Mahidol University, Bangkok, Thailand; 3 Faculty of Medicine Siriraj Hospital, Her Majesty’s Cardiac Center, Mahidol University, Bangkok, Thailand

**Keywords:** Preprocedural and procedural variables, Predict, New-onset conduction disturbances, Transcatheter aortic valve replacement (TAVR)

## Abstract

**Background:**

Conduction disturbances are a common complication after transcatheter aortic valve replacement (TAVR). The aim of this study was to investigate the preprocedural and procedural variables that predict new-onset conduction disturbances post-TAVR (hereafter CD/CDs).

**Methods:**

Consecutive patients who underwent TAVR during December 2009–March 2021 at the Faculty of Medicine Siriraj Hospital, Mahidol University—Thailand’s largest national tertiary referral center—were enrolled. Patients with prior implantation of a cardiac device, periprocedural death, or unsuccessful procedure were excluded. Clinical and electrocardiographic data, preprocedural imaging, including membranous septum (MS) length, and procedural variables, including implantation depth (ID), were analyzed. CD was defined as new left or right bundle branch block, significant intraventricular conduction disturbance with QRS interval ≥ 120 ms, new high-grade atrioventricular block, or complete heart block. Multivariate binary logistic analysis and receiver operating characteristic (ROC) curve analysis were used to identify independent predictors and the optimal ∆MSID (difference between the MS length and ID) cutoff value, respectively.

**Results:**

A total of 124 TAVR patients (mean age: 84.3 ± 6.3 years, 62.1% female) were included. The mean Society of Thoracic Surgeons score was 7.3%, and 85% of patients received a balloon expandable transcatheter heart valve. Thirty-five patients (28.2%) experienced a CD, and one-third of those required pacemaker implantation. The significant preprocedural and procedural factors identified from univariate analysis included intraventricular conduction delay, mitral annular calcification, MS length ≤ 6.43 mm, self-expanding device, small left ventricular cavity, and ID ≥ 6 mm. Multivariate analysis revealed MS length ≤ 6.43 mm (adjusted odds ratio [aOR] 9.54; 95% CI 2.56–35.47; *p* = 0.001) and ∆MSID < 0 mm (adjusted odds ratio [aOR] 10.77; 95% CI 2.86–40.62; *p* =  < 0.001) to be independent predictors of CD. The optimal ∆MSID cutoff value for predicting conduction disturbances was less than 0 mm (area under the receiver operating characteristic curve [AuROC]: 0.896).

**Conclusion:**

This study identified MS length ≤ 6.43 mm and ∆MSID < 0 mm as independent predictors of CDs. ∆MSID < 0 was the strongest and only modifiable predictor. Importantly, we expanded the CD criteria to cover all spectrum of TAVR-related conduction injury to lower the threshold of this sole modifiable risk. The optimal ∆MSID cutoff value was < 0 mm.

*Trial registration*: TCTR, TCTR20210818002. Registered 17 August 2021—Retrospectively registered, http://www.thaiclinicaltrials.org/show/TCTR 20210818002.

## Background

Transcatheter aortic valve replacement (TAVR) is an emerging therapeutic procedure in patients with symptomatic severe aortic stenosis who have intermediate to high surgical risk or who are inoperable [1–5]. Despite the development of the transcatheter heart valve (THV) to decrease periprocedural complications, new-onset conduction disturbances post-TAVR (hereafter collectively referred to as CD/CDs) remain relatively common [6]. The mechanism of CDs may be direct mechanical insult by the THV to conduction tissue, which results in ischemia, edema, or hemorrhage [6]. Based on this mechanism hypothesis, several studies have investigated the predicted risk for permanent pacemaker implantation (PPI) in patients with CDs, but some of the reported predictors were inconsistent among studies [7, 8]. Because of these variations among treatment-related factors, including discrepancies in the indication and timing of PPI, the care decision pathway has not yet been conclusively established in this clinical setting. Moreover, the data are still scarce relative to the clinical outcomes of patients with CDs compared between those treated with and without PPI [9]. It is possible that there may be a single standardized decision pathway that is suitable for use in CD patients.

Accordingly, the aim of this study was to investigate clinical, electrocardiographic, preprocedural imaging, and procedural variables to identify independent factors that predict new-onset conduction disturbances post-TAVR.

## Methods

### Study design and patient population

The study protocol was approved by the Siriraj Institutional Review Board (SIRB) of the Faculty of Medicine Siriraj Hospital, Mahidol University, Bangkok, Thailand (COA no. Si 438/2018). The study protocol conforms to the ethical guidelines of the Declaration of Helsinki, and all included patients gave informed written consent to participate. All consecutive patients who underwent TAVR during December 2009 to March 2021 at Siriraj Hospital were reviewed for inclusion eligibility. Patients with prior implantation of a cardiac device, those who died during the procedure, and who experienced an unsuccessful TAVR procedure were excluded. All patients were assessed by the Siriraj structural heart team, which includes cardiovascular surgeons, a structural interventionist, and structural imaging specialists. The suitability of the procedure, procedural concerns, access site, and transcatheter heart valve (THV) type and size were discussed and determined based on a preprocedural multimodality imaging framework that includes echocardiography, multislice computed tomography (MSCT), and angiography. Additional electrocardiography (ECG), imaging, and procedural data required for this study were analyzed retrospectively.

### Periprocedural conduction monitoring protocol

All TAVR patients at Siriraj Hospital routinely undergo insertion of transvenous temporary pacing in the right ventricle during the procedure, which is then removed in the cardiac intensive care unit. Patients are subsequently monitored at least overnight via telemetry ECG according to Siriraj TAVR postprocedural protocol.

A temporary pacemaker was placed overnight in patients with preexisting conduction disturbance and significant ECG changes during the procedure, which was defined as any new-onset LBBB and/or increase in PR or QRS duration ≥ 20 ms (ms), and/or development of periprocedural transient or persistent heart block. Twelve-lead ECG was recorded pre-TAVR and immediately post-TAVR in all patients until discharge. All ECG data were analyzed by a cardiologist who was blinded to the clinical, preprocedural, and procedural data. Conduction disturbance was defined using the criteria published in the 2018 ACC/AHA/HRS Guideline on the Evaluation and Management of Patients with Bradycardia and Cardiac Conduction Delay [10].

### Preprocedural data analysis

Preprocedural transthoracic echocardiography was performed by echocardiographic specialists who are experienced in the use of echocardiography in structural heart interventions. The definitions of echocardiographic variables [11–17] are shown in Table 1.

**Table 1:** Preprocedural and periprocedural variable definitions

Variables	Definition
*Preprocedural variables*	
3D-derived LVOT area	LVOT area measured by multiplanar reformatting under 3D-guided planimetry method
3D-derived LVOT perimetry	LVOT perimetry measured by multiplanar reformatting under 3D-guided planimetry method
Annular area and perimetry [11]	Annular area and perimetry measured by MSCT using direct planimetry method
Valve oversizing by area (%) [12]	[(THV area in cm2)/(native annular area in cm2) -1] × 100
Valve oversizing by perimetry (%) [12]	[(THV perimetry in mm)/(native annular perimetry in mm) -1] × 100
Eccentricity index	1—(minimum diameter/maximum diameter)
Calcification of the valvular apparatus at aortic cusps and left ventricular outflow tract [13]	Visually graded as none = 0, mild = 1, moderate = 2, and severe = 3
The membranous septum (MS) length (Fig. 1C)	Infra-annular portion of MS measured from coronal view
MAC on MSCT [14]	Presence of dense calcium deposits at the base of mitral leaflets, grade 0 = no MAC, grade 1 = mild MAC involvement affected ≤ ¼ of the annulus, grade 2 = moderate MAC involvement ¼—½ of the annulus, grade 3 severe MAC involvement ≥ ½ of the annulus
*Procedural variables*	
Implantation depth (Fig. 1A, B) [15, 16]	Range between the prosthesis below the aortic annulus plane
ID by frame height below aortic annulus, % (Fig. 1A) [16]	(implantation depth in mm)/(frame height in mm) × 100
∆MSID	Difference between MS length and ID
Small LV cavity	Mentioned small LV cavity during procedure by echocardiographic specialist who performed intraoperative TEE or structural interventionist
PVL	PVL definitions using the VARC-3 consensus [17]

LVOT, left ventricular outflow tract; MAC, Mitral annular calcification; PVL, paravalvular leakage; THV, transcatheter heart valve

After contrast-enhanced MSCT imaging became available at our center, all patients routinely underwent MSCT to evaluate the suitability of TAVR, including the peripheral access vessels; the dimensions of the aortic annulus, which are crucial for THV sizing; and, calcification at the device landing zone. Data acquisition protocols were based on the guidelines published by the Society of Cardiovascular Computed Tomography [11]. The definitions of MSCT variables are shown in Table 1.

### Membranous septum (MS) length and prothesis implantation depth (ID) analysis

**Fig. 1:**
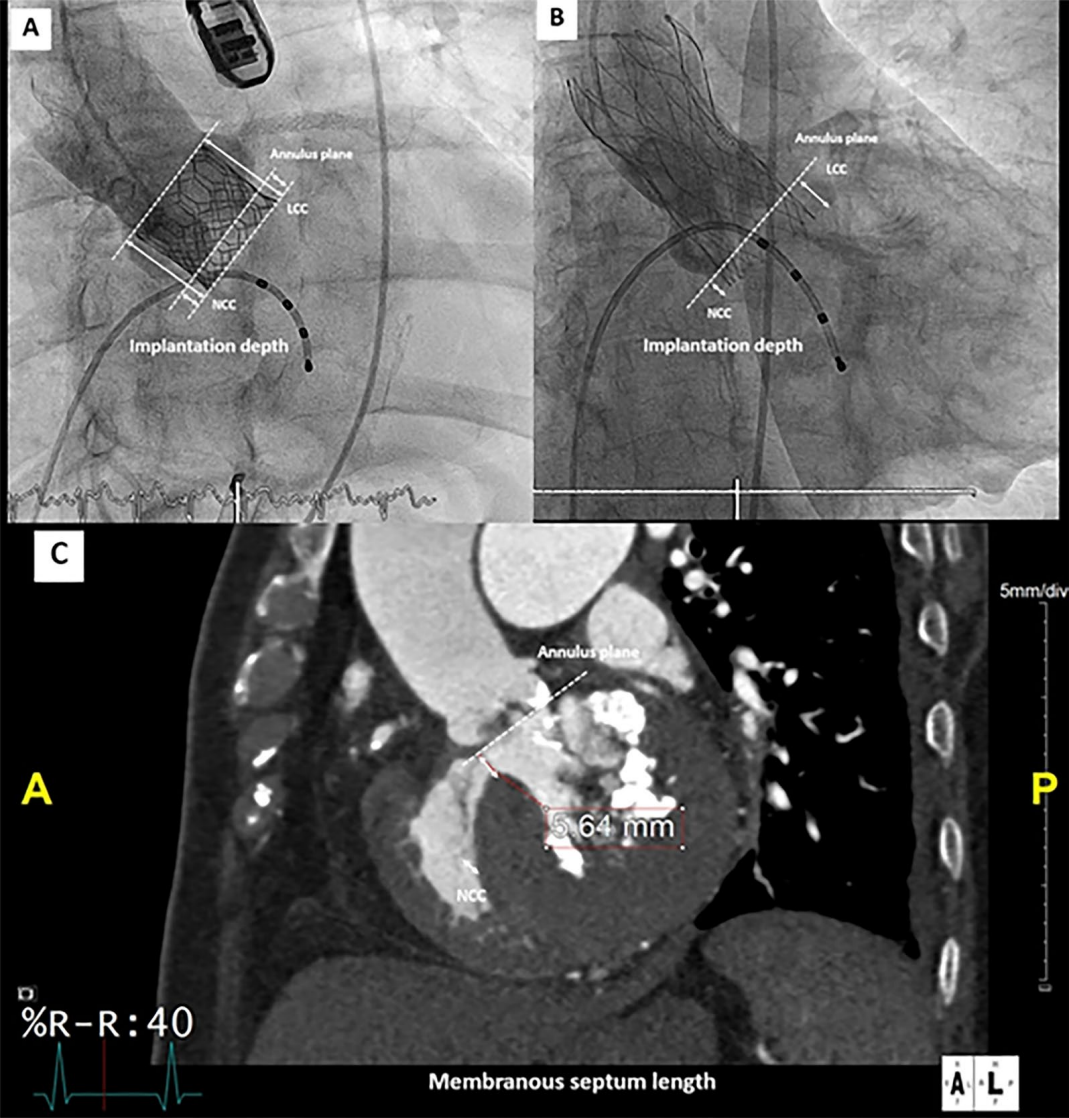
Example of angiography‐derived implantation depth (ID) measurement for balloon-expandable (**A**) and self-expanding (**B**) transcatheter heart valve (THV) as the range between the prosthesis below the aortic annulus plane (short arrows) at the noncoronary cusp to the native aortic annulus plane in the prosthesis in an orthogonal view. ID is expressed as length and percentage of frame height below the aortic annulus. Example of multislice computed tomography (MSCT)-derived coronal membranous septum (MS) length as the distance from the most superior thinnest part of the muscular interventricular septum to the annular plane in the coronal view under the systolic phase (at 40% of the RR interval) (C)

The MS has a supra-annular portion, and an infra-annular portion. The latter plays a role in anatomical recognition of the atrioventricular bundle [18]. In this study, we used the coronal MS length approach, which is defined as the distance from the most superior thinnest part of the muscular interventricular septum to the annular plane in the coronal view under the systolic phase (at 40% of the RR interval) (Fig. 1C). The MS length was not routinely recorded, so it was measured retrospectively by an investigator blinded to all clinical data using Aquarius iNtuition cardiovascular imaging software (TeraRecon, Inc., Durham, NC, USA).

Greater implantation depth (ID) was reported to be an independent risk factor for PPI [6, 15] because the radial force of the prosthesis leads to impinges upon the conduction system. The final post-deployment aortic angiogram in orthogonal view was analyzed offline using IntelliSpace cardiovascular software version 2.3 (Philips Healthcare, Amsterdam, the Netherlands). The depth of implantation was demonstrated as the distance in mm and percentage frame height below the septal site of aortic annulus for both balloon-expandable (Fig. 1A) and self-expanding THVs (Fig. 1B). The difference between MS and ID (∆MSID) was calculated.

### New-onset conduction disturbance post-TAVR (CD) and clinical outcome definitions

New-onset CD post-TAVR (CD) was defined as any conduction disturbance that occurred during the periprocedural TAVR period (procedure and hospitalization period). CDs selected as study endpoints included new complete left and right bundle branch block (LBBB, RBBB), significant intraventricular CD (IVCD) with QRS interval ≥ 120 ms, new high-grade atrioventricular block (HAVB), or complete heart block (CHB). Concerning immediate post-TAVR ECG, significant ECG change was defined as any new-onset LBBB and/or increase in PR or QRS duration ≥ 20 ms and/or development of periprocedural transient or persistent heart block [9]. Late high-degree CDs were recorded if they occurred > 48 h after TAVR or after hospital discharge [9].

The clinical cardiac electrophysiologist made the final decision regarding pacemaker implantation. In order to decrease treatment variation bias, we expanded the criteria for defining a CD to cover all spectrum of TAVR-related the new-onset CD in addition to PPI as the study endpoint.

All patients with a CD were routinely followed-up with clinical, ECG, and echocardiographic assessment for overall assessment, and to evaluate for resolution of the conduction disturbance. Clinical outcomes based on the Valve Academic Research Consortium-3 (VARC-3) consensus document [17] were reviewed using internal and external medical records.

PPI patients were followed-up at the pacemaker clinic at 1 month, 3- to 6-months, and 12 months. Recorded data from pacemaker interrogation were analyzed to determine pacemaker dependency with pacing percentage as the measurement parameter.

### Statistical analysis

Baseline patient characteristics, comorbidities, ECG data, and preprocedural and procedural parameters were compared between the presence or absence of CD. Categorical variables are presented as numbers and percentages. Continuous variables are expressed as mean ± standard deviation (SD) or median and interquartile range (IQR). The Shapiro–Wilk test is used method to test the normality of the continuous data. Continuous data were compared using Student’s t-test (normality) or Mann–Whitney U test (non-normality). Categorical data were compared using chi-square test or Fisher’s exact test. Independent association between CD and prespecified clinical, ECG, preprocedural, and procedural parameters was analyzed using binary logistic regression with forward stepwise multivariate selection. Odds ratios (ORs) with their corresponding 95% confidence intervals (CIs) were estimated. Parameters with a *p*-value ≤ 0.2 in univariate analyses were tested in multivariate analyses with preprocedural and procedural parameters being analyzed separately. A *p*-value of less than 0.05 in multivariate analysis indicated statistical significance. Receiver operating characteristic (ROC) curve analysis was employed to identify the preprocedural and procedural parameters that best predict a CD, and to determine the optimal cut-off value for that/those parameter(s). All statistical analyses were performed using SPSS Statistics for Windows, version 23.0 (SPSS, Inc., Chicago, Ill., USA).

## Results

During December 2009 to March 2021, 132 patients underwent TAVR. After exclusion of patients with implantation of cardiac device at baseline (n = 6, 4.5% of all patients), or intraoperative death (n = 2, 1.5% of all patients), the remaining 124 patients were enrolled. Of those, 35 (28.2%) patients developed CDs, including 19 (54.3%) new-onset LBBB, 13 (37.1%) new-onset significant CD, and 3 (8.6%) new-onset high-grade AV block. The overall rate of permanent pacemaker implantation was 9.7% (12 of 124 cases). Preprocedural and procedural clinical and ECG characteristics are shown in Tables 2, 3 and 4.

**Table 2:** Baseline demographic, clinical, and electrocardiographic characteristics

Parameters	All patients N = 124	With CD N = 35	No CD N = 89	*p* value
*Clinical characteristics*				
Age (years)	84.3 ± 6.3	84.5 ± 6.3	84.2 ± 6.4	0.806
Female	77 (62)	22 (63)	55 (62)	0.913
STS PROM (%)	7.3 ± 5.1	7.4 ± 4.5	7.3 ± 5.3	0.877
NYHA III or IV	69 (56)	19 (54)	50 (56)	0.848
Diabetes mellitus	46 (37)	14 (40)	32 (36)	0.675
CAD	84 (68)	24 (67)	60 (67)	0.901
Prior MI	30 (24)	9 (26)	21 (24)	0.804
Prior PCI	51 (41)	10 (29)	41 (46)	0.075
CABG	17 (13.7)	6 (17)	11 (12)	0.486
Cerebrovascular disease	11 (9)	5 (14)	6 (7)	0.184
Peripheral artery disease	21 (17)	4 (11)	17 (19)	0.305
COPD	8 (7)	2 (6)	6 (7)	0.834
Hypertension	116 (94)	33 (94)	83 (93)	0.834
Paroxysmal or persistent AF	32 (26)	10 (29)	22 (25)	0.659
eGFR (ml/min)	45.1 ± 22.3	45.4 ± 23.1	45.0 ± 22.1	0.916
History of syncope	25(20)	8(23)	17(19)	0.639
Beta-blocker therapy	54(44)	12(34)	42(47)	0.192
*ECG characteristics*				
Baseline AF	20(16)	5(14)	15(17)	0.726
Baseline heart rate (beats/min)	73.3 ± 13.2	71.9 ± 14.1	73.8 ± 12.9	0.481
Baseline PR interval (ms)	187.9 ± 36.8	181.9 ± 38.1	190.2 ± 36.3	0.303
Baseline QRS duration (ms)	104.8 ± 23.2	104.2 ± 19.5	105.1 ± 24.6	0.863
First-degree AVB	32(26)	7(20)	25(28)	0.354
Left bundle branch block	4(3)	0(0)	4(5)	0.202
Right bundle branch block	21(17)	6(17)	15(17)	0.969
Left anterior fascicular block	5(4)	1(3)	4(5)	0.677
Left posterior fascicular block	3(2)	1(3)	2(2)	0.842
Significant IVCD	21(17)	11(31)	10(11)	**0.007**

Data presented as frequency and percentage or mean ± standard deviation

A *p* value < 0.05 indicates statistical significance

AF, atrial fibrillation; AVB, atrioventricular block; CABG, coronary artery bypass surgery; CAD, coronary artery disease; CD, conduction disturbances; COPD, chronic obstructive pulmonary disease; eGFR, estimated glomerular filtration rate; IVCD, intraventricular conduction delay; MI, myocardial infarction; MS, membranous septum; NYHA, New York Heart Association; PCI, percutaneous coronary intervention; STS-PROM, Society of Thoracic Surgeons predicted risk of mortality

**Table 3:** Analysis for significant preprocedural predictors of new-onset conduction disturbance post-TAVR

Parameters	All patients N = 124	With CD N = 35	No CD N = 89	*p* value
*Pre-procedural echocardiography*				
Aortic valve area (cm^2^)	0.75 ± 0.22	0.76 ± 0.19	0.74 ± 0.23	0.782
Mean AV pressure gradient (mmHg)	50.7 ± 16.1	47.6 ± 11.14	51.8 ± 18.2	0.201
LVEDV (ml)	70.1 ± 28.5	63.46 ± 21.03	72.67 ± 30.69	0.106
LVEF (%)	63.8 ± 14.7	66.9 ± 10.42	62.66 ± 15.95	0.143
LV mass index (g/m^2^)	158.6 ± 43.7	142.1 ± 25.51	165.36 ± 47.7	0.008
LVOT area (mm^3^)	400.9 ± 61.6	408.4 ± 59.9	397.7 ± 62.4	0.417
LVOT perimetry (mm)	75.0 ± 8.6	75.1 ± 8.8	75 ± 8.7	0.987
*Pre-procedural MSCT*				
Maximal annular diameter (mm)	25.3 ± 2.4	25.75 ± 2.7	25.03 ± 2.29	0.169
Minimal annular diameter (mm)	20.3 ± 1.9	20.48 ± 1.9	20.15 ± 1.92	0.431
Eccentricity index	0.196 ± 0.07	0.20 ± 0.07	0.19 ± 0.07	0.571
Annular area (mm^3^)	400.7 ± 61.1	406.4 ± 60.15	398.3 ± 61.7	0.527
Oversizing by annulus (%)	4.02(-0.42–11.13)	3.90(-1.67–9.80)	4.31(0.58–11.5)	0.190
Annular perimetry (mm)	72.9 ± 5.5	73.6 ± 5.6	72.7 ± 5.5	0.487
Oversizing by Perimetry (%)	-0.15(-2.98–2.79)	-0.75(-3.27–2.92)	0.03(-2.75–2.79)	0.338
Severe AV calcification	35(34.3)	8(25.8)	27(38.0)	0.232
Presence of LVOT calcification	15(14.6)	5(15.6)	10(14.1)	0.837
Moderate MAC	18(14.5)	10(28.6)	8(9)	0.005
MS length (mm)	7.5 ± 2.7	5.6 ± 2.1	8.4 ± 2.5	< 0.001

Data presented as frequency and percentage, mean ± standard deviation or median (interquartile range)

A *p* value < 0.05 indicates statistical significance

AV, Aortic valve; CD, conduction disturbances; LV, left ventricle; LVEF, left ventricular ejection fraction; LVEDV, left ventricular end-diastolic volume; LVOT, left ventricular outflow tract; MAC, Mitral annular calcification; MS, Membranous septum

**Table 4:** Analysis for significant procedural predictors of new-onset conduction disturbance post-TAVR

Parameters	All patients N = 124	With CD N = 35	No CD N = 89	*p* value
*Procedural characteristics*				
Procedural time (min)	95.2 ± 43.4	89.7 ± 33.6	97.5 ± 46.9	0.392
Fluoroscopy time (min)	21.1 ± 8.9	23.4 ± 9.5	20.2 ± 8.7	0.106
Contrast (ml)	103.6 ± 52.9	108.3 ± 53.1	101.6 ± 53.2	0.560
Transapical access	32(26)	5(14)	27(30)	0.066
Self-expanding THV	18(15)	10(29)	8(9)	0.005
Edward SAPIEN 3	80(65)	23(66)	57(64)	0.861
Balloon Predilation	72(58)	19(54)	53(59)	0.593
Balloon Postdilation	47(38)	17(49)	30(34)	0.125
Final PVL ≥ 1 +	51(41)	13(37)	38(43)	0.572
Small LV cavity	19(15)	11(31)	8(9)	0.002
ID (mm)	4.9 ± 1.9	6.2 ± 2.4	4.4 ± 1.3	< 0.001
ID by frame height below aortic annulus (%)	24.2 ± 6.5	28.9 ± 7.3	22.9 ± 5.7	< 0.001
∆MSID (mm)	2.6 ± 3.6	-4.02 ± 2.73	0.754 ± 3.1	< 0.001
ID > MS	22(24)	17(63)	5(8)	< 0.001
*Postprocedural ECG*				
Significant PR change	11(9.7)	7(22.2)	4(5.3)	0.011
Significant QRS change	34(27.4)	31(85.7)	3(3.4)	< 0.001
Significant ECG change	41(33.1)	34(97.1)	7(7.9)	< 0.001
*Postprocedural outcome*				
Length of stay (days)	5(3–9)	5(4–9)	5(3–9.5)	0.910
All cause periprocedural mortality	6(4.8)	2(5.7)	4(4.5)	1.000
All cause early mortality	4(3.2)	0(0)	4(4.5)	0.576

Data presented as frequency and percentage or mean ± standard deviation

A p-value < 0.05 indicates statistical significance

∆MSID, difference between MS length and ID; CD, conduction disturbances; PVL, paravalvular leakage; ID, implantation depth; LV, left ventricle; THV, transcatheter heart valve; MS, membranous septum

### Preprocedural predictors of CDs

Clinical and ECG characteristics are presented in Table 2. The average age of patients was 84 years, and there were 77 (62%) women). The mean Society of Thoracic Surgeons (STS) Predicted Risk of Operative Mortality score was 7.3%. The baseline clinical characteristics of patients with and without CDs were similar for age, gender, STS score, presenting symptoms, and comorbidities (Table 2). There were no significant differences in baseline ECG characteristics between groups, except for prior significant intraventricular conduction delay (IVCD), which was significantly more frequent in patients with CDs (31% vs. 11%, *p* = 0.007).

Regarding preprocedural predictors (Table 3), patients who developed CDs were more likely to have low left ventricular (LV) mass index by echocardiography (142.1 ± 25.5 vs. 165.4 ± 47.7 g/m^2^, *p* < 0.001), more likely to have moderate mitral annular calcification (MAC) (28.6% vs. 9.0%, *p* = 0.005), and shorter MS length by MSCT (5.6 ± 2.1 vs. 8.4 ± 2.5 mm, *p* < 0.001).

Concerning the annular parameters, there was no statistically significant difference in the degree of oversizing between groups. There was also no statistically significant difference in the severity of valvular calcification, presence of left ventricular outflow tract (LVOT) calcification, or eccentricity index between groups.

Preprocedural characteristics, including echocardiography and MSCT, significantly associated with the occurrence of CDs (Table 3). Patients with CDs had significantly lower LV mass index (142.1 ± 25.51 vs. 165.36 ± 47.7 g/m^2^, *p* < 0.001) and shorter MS length (5.6 ± 2.1 vs. 8.4 ± 2.5 mm, *p* < 0.001).

### Procedural predictors of CDs, and postprocedural ECG

The procedural characteristics of patients with and without CDs are displayed in Table 4. Patients with CDs significantly more frequently received the self-expandable THV Portico TAVR system (St. Jude Medical, St. Paul, MN, USA) (29% vs. 9%, *p* = 0.005), were more likely to have a small LV cavity during the TAVR procedure (31% vs. 9%, *p* = 0.002), and more likely to have a deeper implantation depth (6.2 ± 2.4 vs. 4.4 ± 1.3 mm, *p* < 0.001).

### Receiver operating characteristic curve analysis to identify the most predictive parameter

We used receiver operating characteristic (ROC) curve analysis for determined the best cut-off value for the statistical significance of variables, and validity was assessed by the area under the curve (AUC). The optimal MS length cutoff value for predicting CDs was ≤ 6.43 mm (area under the receiver operating characteristic curve [AUC]: 0.822) and the optimal ∆MSID cutoff value for predicting CDs was < 0 mm (area under the receiver operating characteristic curve [AUC]: 0.896).

The optimal ∆MSID cut-off was determined to be < 0 mm, or when the ID was more than the MS length. Concerning diagnostic performance, this cutoff for predicting CDs showed a sensitivity of 62.96% (95% CI 42.37–80.60), a specificity of 92.31% (95% CI 82.95–97.46), a positive predictive value of 77.27% (95% CI 58.26–89.23), and a negative predictive value of 85.71% (95% CI 78.50–90.79).

### Analysis for factors that independently predict CDs

Preprocedural and procedural variables were separately analyzed using univariate logistic regression to identify potential predictors of CDs (Table 5). Variables with a *p*-value ≤ 0.2 were then entered into multivariate logistic regression analysis. The preprocedural variables found to be associated with CDs included preexisting IVCD, presence of MAC ≥ 2+, and MS length ≤ 6.43 mm. The procedural factors found to be correlated with CDs were self-expanding THV, ∆MSID < 0 mm, and small LV cavity during the procedure. Transapical access showed low association with CDs. Other variables that was previously report [8] in predicting CDs such as pre-existing LBBB, pre-existing RBBB, balloon predilation, balloon postdilation, prosthesis oversizing, and Edwards SAPIEN-3 valve were also included into analysis. In combined multivariate logistic regression analysis, MS length ≤ 6.43 mm (adjusted odds ratio [aOR] 9.54; 95% CI 2.56–35.47; *p* = 0.001) and ∆MSID < 0 mm (adjusted odds ratio [aOR] 10.77; 95% CI 2.86–40.62; *p* =  < 0.001) to be independent predictors of CD.

**Table 5:** Univariate and multivariate analysis to identify preprocedural and procedural predictors of new-onset conduction disturbance post-TAVR

Predictors	Univariate analysis		Multivariate analysis	
	Odds ratio (95% CI)	*p* value	Odds ratio (95% CI)	*p* value
*Preprocedural aspects*				
Pre-existing IVCD	3.62 (1.37–9.56)	0.009		
Presence of MAC ≥ 2 +	4.05 (1.44–11.37)	0.008		
MS length ≤ 6.43 mm	13.55 (4.46–41.17)	< 0.001	9.54 (2.56–35.47)	0.001
*Procedural aspects*				
Self-expanding THV	4.05 (1.44–11.37)	0.008		
∆MSID < 0 mm	20.40 (6.14–67.8)	< 0.001	10.77 (2.86–40.62)	< 0.001
Deep implantation depth ≥ 6 mm	17.79 (6.02–52.57)	< 0.001		
Small LV cavity	4.64 (1.68–12.85)	0.003		

A *p* value < 0.05 indicates statistical significance

Adjusted odds ratio for pre-existing IVCD, presence of MAC ≥ 2+, MS length ≤ 6.43 mm, self-expanding THV, ∆MSID < 0, deep implantation depth ≥ 6 mm, small LV cavity, pre-existing LBBB, pre-existing RBBB, balloon predilation, balloon postdilation, prosthesis oversizing, and Edwards SAPIEN-3

CD, conduction disturbances; CI, confidence interval; OR, odds ratio; IVCD, intraventricular conduction delay; MAC, Mitral annular calcification; THV, transcatheter heart valve; MS, membranous septum; ∆MSID, difference between MS length and ID; LV, left ventricle

### Timing of and indications for pacemaker implantation

Among the 35 patients with CDs, PPI was performed in 12 patients (34.3% of patients with CDs). Indications for PPI included 7 (58%) new-onset LBBB, and 5 (42%) HAVB or CHB. New-onset LBBB patients who received PPI had a median QRS duration of 156 ms (interquartile range [IQR] 149.25–160.75). Patients who did not undergo PPI had a median QRS duration of 152.5 ms (IQR 134.5–157.5). Patients underwent PPI at a median 6 days (IQR 2.25–13.5). Only two CD patients were diagnosed as late high-degree CD. Those patients presented with AF and slow AV conduction at the 2- and 3-month follow-ups, respectively. No patient without new-onset CD developed any late high-degree CDs during the follow-up period.

### Follow-up of patients with CDs

**Fig. 2:**
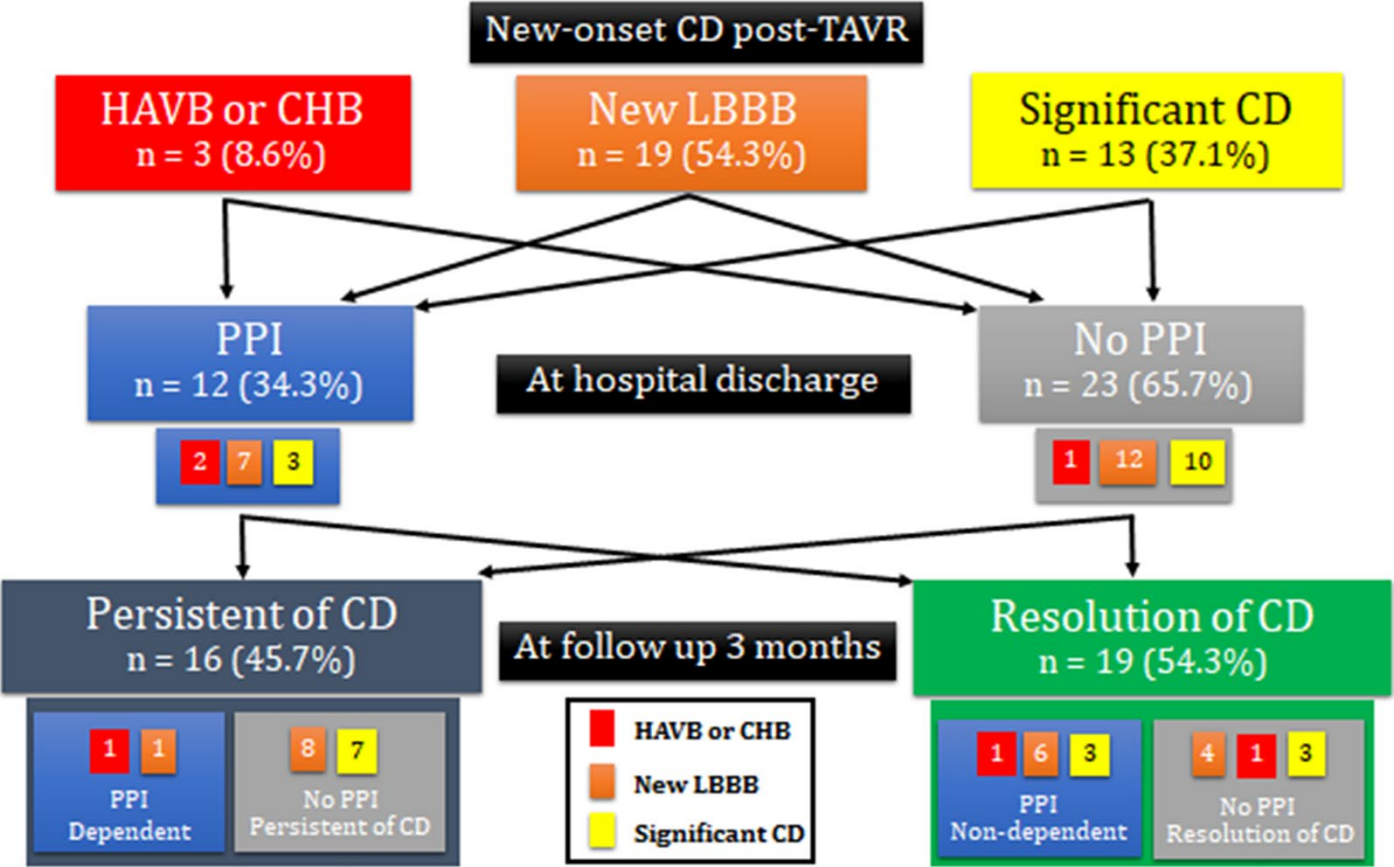
Diagram showing follow-up data of patients with new-onset conduction disturbances post-transcatheter aortic valve replacement; HAVB high-grade AV block, CHB complete heart block, LBBB left bundle branch block, CD conduction disturbances, PPI permanent pacemaker implantation, TAVR transcatheter aortic valve replacement

Our analysis of follow-up data from patients with CDs is shown in Fig. 2. More than half of patients with CDs had resolution of conduction disturbances by the 3-month follow-up. Most CD cases resolved by 1 month after TAVR. Eighty-five percent of patients with PPI were pacemaker independent. Only 2 (16%) patients with PPI were pacemaker dependent at the 3-month follow-up. One of those two patients had complete heart block immediately after THV deployment, which persisted until hospital discharge and throughout follow-up. The other had atrial fibrillation (AF) with new LBBB (QRS duration: 160 ms). The electrophysiologist initially felt that PPI was not indicated; however, that decision was reversed due to the presence of late high-degree conduction disturbances presenting as AF with bradycardia.

## Discussion

The present study investigated preprocedural and procedural variables to predict a new-onset conduction disturbance post-TAVR (CD/CDs). The main findings of this study are, as follows: (1) The prevalence of CD development was 28.2%, and PPI was required in 9.7%.; (2) The preprocedural characteristics prior IVCD, MAC, lower LV mass index, and MS length $\le$ 6.43 mm were more likely to be present in patients with a CD; (3) Procedural characteristics of the use of a self-expandable TAVR, smaller LV cavity during the procedure, and ID > 6 mm were also more commonly present in patients with a CD; (4) Preprocedural MS length and postprocedural difference between MS length and ID (∆MSID) was found to be the strongest predictors of a CD; (5) An ∆MSID cut-off value of < 0 mm was found to most strongly predict a CD. Importantly and different from any previous study, we expanded the criteria for defining a CD to cover all spectrum of TAVR-related conduction injury to lower the threshold of this only modifiable risk factor.

This study found a CD rate of 28.2%, which corresponds with the previously reported incidence of new LBBB after TAVR (range 20% to 34%) [8, 19]. One-third of our patients with CD were treated by PPI. We broadened our study CD endpoint to include new-onset complete LBBB and/or RBBB, significant IVCD (QRS duration ≥ 120 ms), and new-onset HAVB or CHB for cover the entire spectrum of TAVR-related conduction injury.

Our study categorized variables into preprocedural and procedural predictors, and we included many parameters that were reported to be associated of conduction disturbances post-TAVR. This study included clinical, electrocardiographic, echocardiographic, MSCT, and procedural data for analysis. Transapical access was reported to be less likely associated with new-onset CD post-TAVR due to the use of a balloon expanding device [20]. Parameters, including intraventricular conduction delay, mitral annular calcification, MS length ≤ 6.43 mm, lower LV mass index, small LV cavity, use of self-expanding device, ID ≥ 6 mm, were analyzed for their ability to significantly/independently predict CD. We also included other variables that was previously report [8] in predicting CDs in the analysis. The results of our multivariate analysis revealed MS length ≤ 6.43 and ∆MSID < 0 as independent predictors of CDs. The lower border part of the MS is the typical location of the atrioventricular bundle [18]. Shorter MS also correlates with high position or less ventricular position of the anatomical atrioventricular bundle. MS length is easily measured with good reproducibility in the coronal plane [21]. Mauri, et al. first described the usefulness of angiographic implantation depth, device landing zone calcium volume, and baseline RBBB as independent predictors of the need for PPI post-TAVR [22]. Maeno, et al. identified non-coronary cusp device landing zone calcium volume, RBBB, and ∆MSID as synergistic PPI predictors [23]. The usefulness of calcium in device landing zone and implantation depth in predicted PPI in balloon expanding device have been demonstrated [24]. Tretter, et al. analyzed gross anatomical variation in the aortic root, such as the distance from the virtual basal ring to the inferior margin of the MS, relative to correlation with PPI or LBBB. They found TAVR valve type and ID to be the primary procedural factors associated with conduction damage regardless of gross anatomical variation [25]. Concerning TAVR valve type, the Sapiens 3 and Accurate Neo valves were reported to be associated with the lowest PPI rate, followed by the Evolut and Portico valves [26]. Jilaihawi, et al. proposed an anatomically-guided minimized depth during the MS approach for implantation of a self-expanding TAVR [15]. Chen, et al. reported an ∆MSID of 3.2 mm to be the optimal cutoff point for predicting a CD when using a self -expanding device [21]. For balloon expanding TAVR, mean ∆MSIDs of − 2.5 ± 2.4 mm [23], 0.5 ± 4 mm [24], and − 1.7 ± 1.5 mm [27] were reported in patients with PPMI or LBBB. Miki, et al. reported that an ∆MSID cut-off value of -0.7 mm had an AuROC of 0.85, sensitivity of 0.78, and specificity of 0.82 (*p* < 0.001). That group defined a CD as an atrioventricular block that required PPI or new-onset complete LBBB. Because the ∆MSID was found to be the only modifiable predictor, we expanded our definition of CD to include PPI, new onset-LBBB, onset-RBBB, significant IVCD (QRS duration ≥ 120 ms), and new-onset HAVB or CHB for comprehensively cover the spectrum of TAVR-related conduction injury. In our study, an ∆MSID cut-off value of < 0 mm was found to be the optimal cutoff point for predicting a CD (AuROC: 0.896).

Factors, such as baseline RBBB, amount of calcium at the device landing zone, and MS length, are not modifiable. We, therefore, lowered the threshold of the only modifiable predictor (∆MSID) to cover the entire spectrum of TAVR-related conduction injury, which has the effect of lowering the risk of PPI. Using an ∆MSID cut-off value of < 0 mm (i.e., attempt to deploy the THV at an ID less than the MS length) is the best way to minimize the of CDs.

Many techniques have introduced to minimize implantation depth. Cusp-Overlap view strategy recommended for self-expandable valve implantation [28, 29]. For balloon-expandable SAPIEN-3 (S3) valve, the systematic approach to high deployment technique of SAPIEN-3 valve reduced 30-day permanent pacemaker rates from 13.1% to 5.5% [30]. However, this systemic approach to high implantation may constraint future coronary access in certain anatomical subtypes such as low coronary height, narrow coronary sinus, low and narrow sinotubular junction. The risk of coronary obstruction, future coronary access and new-onset CDs post-TAVR can be balanced by knowing anatomy and its predictors.

This study confirmed the usefulness of immediate postprocedural 12-lead ECG as a screening tool for significant ECG change, including new-onset LBBB and/or increase in PR or QRS duration ≥ 20 ms and/or development of periprocedural transient or persistent heart block, as an indicator of the occurrence of new-onset of conduction disturbances [9, 31]. We also reported a late conduction disturbance rate at 1.6%, which is similar previous reports (range 2% to 7%) [6, 32]. More specifically, we had only 1 patient with no significant peri- or immediate postprocedural ECG change that developed late high-degree conduction disturbances with AF with bradycardia during follow-up.

The rate of pacemaker dependence reported in previous studies ranged from one- to two-thirds of patients during follow-up [33–35]. New-onset LBBB post-TAVR resolved in 45% (range 35–56%) in 30 days [6]. Our study found a lower rate of pacemaker dependency (16%) with a similar rate of conduction disturbance resolution (55%). This difference between studies may be explained by a higher unnecessary PPI rate. The most common indication for PPI according to the judgment of our electrophysiologist was new-onset LBBB. Further study is needed to identify the appropriate indication(s) for PPI in this clinical setting.

### Limitations

This study has some mentionable limitations. First, since this study was conducted in a single-center tertiary care setting, center-specific biases cannot be excluded. Second, our study’s retrospective design renders it vulnerable to missing or incomplete data. Third, because patients were enrolled across a + 10-year study period, chronological bias due to improvements in skill and technique can also not be ruled out. However, this study includes homogenous population, structural operator, adherence to a constant clinical evaluation and follow-up, and consistent quality of cardiac imaging. Fourth, although the final decision regarding PPI was made by a clinical cardiac electrophysiologist, variation in treatment, especially the indication for and timing of PPI in new-onset LBBB, is a concern due to the lack of a consensus guideline. Fifth, the number of cases with new-onset LBBB was too small to investigate for the optimal QRS duration cut-off value for use as an indication for pacemaker implantation. Sixth, our study should be interpreted with caution due to relatively small sample size. Seventh, even though the reproducibility of measurements of the MS were good, the MS length may not be measurable at the minimum resolution of MSCT, especially in patients with a shorter MS length. Lastly, Due to the lack of calcium volume analysis in standard MSCT protocol imaging in the aortic-valvular complex, qualitative assessment of calcium volume could not be performed.

## Conclusions

The results of this study revealed preprocedural MSCT-derived MS length and procedural ID to be independent predictors of new-onset conduction disturbance post-TAVR. An ∆MSID cut-off value of < 0 mm was found to optimally predict a CD. Importantly and in contrast to previous studies that investigated and reported a limited criteria for defining a CD, we investigated the full spectrum of possible causes of CD, including PPI, new onset-LBBB, onset-RBBB, significant IVCD (QRS duration ≥ 120 ms), and new-onset HAVB or CHB, because the ∆MSID was found to be the only modifiable predictor. This insight encourages routine deployment of the THV at an ID less than the MS length.

## Data Availability

The datasets used and/or analyzed in this study have been deidentified and available from the corresponding author on reasonable request. Identifying/confidential patient data should not be shared.

## Abbreviations

CD: Conduction disturbances
CHB: Complete heart block
HAVB: High-grade AV block
ID: Implantation depth
IVCD: Intraventricular conduction disturbance
LBBB: Left bundle branch block
MS: Membranous septum (MS) length
∆MSID: Difference between MS and ID
MSCT: Multi-slice computed tomography
PPI: Permanent pacemaker implantation
RBBB: Right bundle branch block
TAVR: Transcatheter aortic valve replacement
